# Role of data uncertainty when identifying important areas for biodiversity and carbon in boreal forests

**DOI:** 10.1007/s13280-023-01908-2

**Published:** 2023-09-01

**Authors:** Heini Kujala, Francesco Minunno, Virpi Junttila, Ninni Mikkonen, Annikki Mäkelä, Raimo Virkkala, Anu Akujärvi, Niko Leikola, Risto K. Heikkinen

**Affiliations:** 1 Finnish Natural History Museum, University of Helsinki, (Pohjoinen Rautatiekatu 13), P.O. Box 17, 00014 Helsinki, Finland; 2 Department of Forest Science, University of Helsinki, P.O. Box 27, 00014 Helsinki, Finland; 3 Finnish Environment Institute Syke, Latokartanonkaari 11, 00790 Helsinki, Finland; 4 Department of Geosciences and Geography, University of Helsinki, Helsinki, Finland

**Keywords:** Biodiversity conservation, Carbon sequestration, Forest growth model, Model uncertainty, Spatial conservation planning, Species distribution modelling

## Abstract

Forest conservation plays a central role in meeting national and international biodiversity and climate targets. Biodiversity and carbon values within forests are often estimated with models, introducing uncertainty to decision making on which forest stands to protect. Here, we explore how uncertainties in forest variable estimates affect modelled biodiversity and carbon patterns, and how this in turn introduces variability in the selection of new protected areas. We find that both biodiversity and carbon patterns were sensitive to alterations in forest attributes. Uncertainty in features that were rare and/or had dissimilar distributions with other features introduced most variation to conservation plans. The most critical data uncertainty also depended on what fraction of the landscape was being protected. Forests of highest conservation value were more robust to data uncertainties than forests of lesser conservation value. Identifying critical sources of model uncertainty helps to effectively reduce errors in conservation decisions.

**Supplementary Information:**

The online version contains supplementary material available at 10.1007/s13280-023-01908-2.

## Introduction

Forests play a central role in the global efforts of halting biodiversity loss and mitigating climate change. In the boreal region, forests account for > 30% of global forest carbon stock and 20% of the annual global forest carbon sink (Pan et al. 2011; Gauthier et al. 2015). Boreal forests comprise one-third of global forest cover, providing habitat for a large number of species (IPBES 2019), yet nearly two-thirds of these forests are under economic use (Gauthier et al. 2015). Intensive forestry and clear-cut logging reduces habitat available for several forest-dwelling species (IPBES 2019; Mönkkönen et al. 2022), and although some early succession species may benefit from harvesting, regenerated stands hold less biological and structural diversity than those originating from natural disturbances (Gauthier et al. 2015). Forest management can increase their carbon sequestration but the benefits of this depend on whether the harvested wood is used for products that prevent the carbon being re-released back to atmosphere in long (e.g. hardwood) or short (e.g. pulp, paper) term (Soimakallio et al. 2016). Clear cutting also decreases the carbon stocks of forests and increases emissions from decomposing harvest residues (Kolari et al. 2004; Goulden et al. 2011).

In Finland, situated in the boreal region, high and low productive forests combined cover about 22.7 million hectares (75%) of the land area (Vaahtera et al. 2021). Around 90% of these are under even-aged rotation forestry, predominantly coniferous managed forests, while the rest are protected. The average size of clear-cut, where no or only a small number of trees are left as seed or retention trees, is 1.76 ha, and afterwards stands are re-planted or allowed to regenerate naturally, and the rotation between final fellings is on average 80–100 years (Kniivilä et al. 2020). The long history of logging and active management has significantly altered the age structure and functional heterogeneity of the Finnish forests (Gauthier et al. 2015; Korhonen 2021), decreasing the area of old-growth forests, number of large trees and the volume of dead wood (Mönkkönen et al. 2022). These large-scale alterations have led to declines in forest biodiversity: 11% of forest species and 76% of the forest habitat types are threatened (Hyvärinen et al. 2019; Kontula and Raunio 2019). Currently, forest heterogeneity is predominantly driven by forestry treatments, forest site type (describing site fertility and aridity) and climate.

The synergistic benefits of forest conservation for both biodiversity and climate change mitigation are increasingly recognised in international and national policies, such as the EU 2030 Biodiversity Strategy, although how much carbon sinks should be emphasised over carbon storages in these policies is still debated (Soimakallio et al. 2016; Pukkala 2018). Nevertheless, through the identification of forests important for both biodiversity and carbon, forest conservation can be strategically targeted to areas that support meeting both policy targets cost-efficiently. In recent years, studies have demonstrated the use of spatial prioritisation (optimisation) tools (Moilanen et al. 2009) for identifying co-benefits and trade-offs between biodiversity and carbon services at various spatial scales (e.g. Forsius et al. 2021; Jung et al. 2021).

Such optimisation exercises require large-scale spatial data. Both biodiversity and carbon data are commonly created using models that estimate the habitat suitability of forest stands for different species and the amount of carbon stored in or sequestered by them (Forsius et al. 2021; Miettinen et al. 2021). As all model estimates contain uncertainty, the goodness of any spatial prioritisation result is inherently dependent on the accuracy of the input data that underpins it. The effects of model uncertainty on the estimated values have been extensively studied (e.g. Barry and Elith 2006; Mäkelä et al. 2020), but how these uncertainties impact spatial optimisation results is less well understood. Recent research has quantified how the impact of data uncertainties on prioritisation results depends on the alteration made, characteristics of the input data and the total data pool that is used to produce the priority result (Kujala et al. 2018a, b). The first-line improvements of modelled input data should focus on uncertainties that, when resolved, change the prioritisation result the most.

Here, we explore the impact of different data uncertainties on conservation prioritisation within a real-world forest conservation context. We look at how the uncertainty in forest variable estimates affects modelled distributions of biodiversity and carbon patterns, and how this in turn introduces variability in a hypothetical conservation plan. We use several features of carbon (size of carbon sinks, amount of carbon stored in trees, ground vegetation and soil) and biodiversity to explore their co-occurrences and role in multi-objective conservation planning, and three time periods from present to 2050 to understand their dynamics through time. We purposefully investigate the data uncertainties in the absence of other major drivers, such as forest harvesting and climate change, so as to exclude their impact. Our purpose is not to identify what areas to protect, but to understand how this decision is impacted by data uncertainties. In particular, we explore whether forest conservation strategies are more sensitive to uncertainties associated with biodiversity vs carbon estimates, and which of the individual sources of uncertainty are most important to solve so to effectively reduce potential errors in spatial conservation decisions.

## Materials and methods

### Overview of the analysis design

**Fig. 1:**
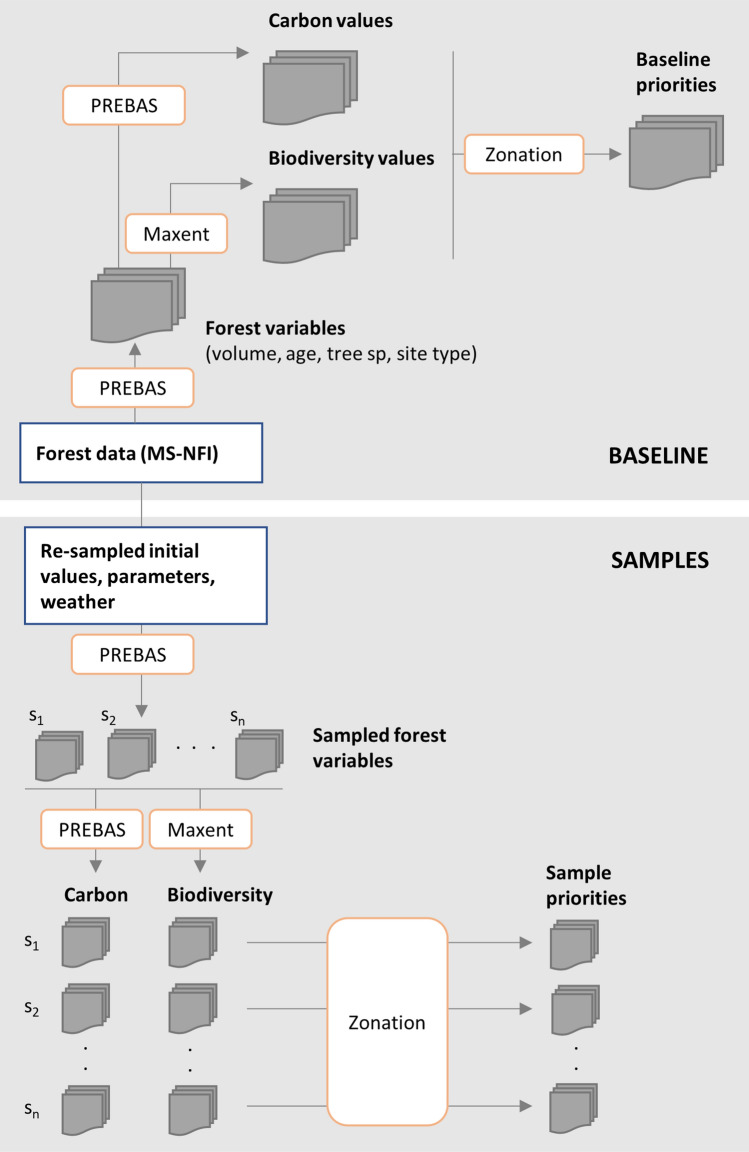
Analysis design showing the different modelling steps and main methodological tools used. Data on the original state of forests came from the multi-source national forest inventories (MS-NFI). The carbon-balance-based stand growth and gas exchange model PREBAS were used to first simulate forest growth and stand characteristics (forest variables) and then to estimate the size of carbon storage and sinks in forest stands (carbon values). The machine learning tool Maxent was used to model habitat suitability of forests for six bird species (biodiversity values), while also accounting for other non-forest variables (climate, land-use types). Finally, Zonation was used to identify potential priority areas for forest conservation. The baseline forest variables were simulated using existing forest data (MS-NFI). For the samples (*s*_*1*_, *s*_*2*_, …, *s*_*n*_), the PREBAS simulation was iteratively repeated while introducing different sources of uncertainty

We used the following approach in this study. First, using measurements from forest stands and a mechanistic forest growth model, we created spatial data for several forest attributes (structural variables, site type and mean age, hereafter called baseline forest variables, see “Forest and carbon data” section). These baseline estimates were then used to model the presence of carbon and biodiversity values across the study area (baseline carbon values, “Forest and carbon data” section and baseline biodiversity values, “Biodiversity data” section), and to identify most important forest areas for conservation at each time period (baseline priorities, “Spatial prioritisation” section) (Fig. 1). Next, we iteratively re-simulated 50 realisations of the forest variables (called sample forest variables, “Sample iterations” section). From each sample, we re-created the maps of carbon and biodiversity data and the respective conservation plan, keeping all other environmental variables constant. This resulted in 50 conservation plans (sample priorities). We measured the variability in the sample model outputs and used a canonical correlation analysis to understand how uncertainty in each forest variable and biodiversity and carbon value affects the final conservation plan (“Quantification of uncertainty” section). In next sections, we describe each analysis step and the data and methods used in more detail.

### Study area

**Fig. 2:**
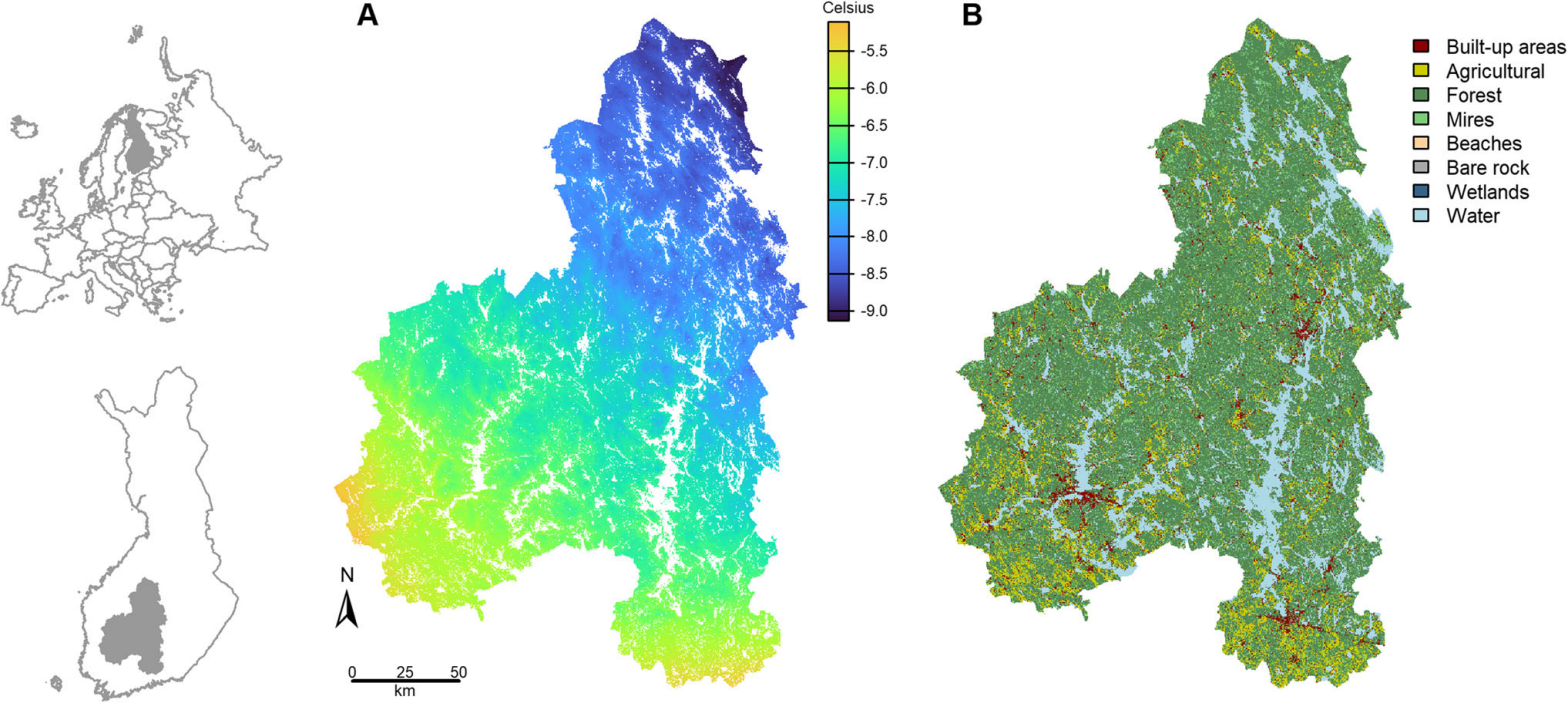
Study area and its location in Finland, Europe. Maps **A** and **B** show the mean January temperature and major land-use categories, respectively

The study area is located in Central Finland, Europe (Fig. 2). The area covers 41 503 km^2^, of which ~ 6 500 km^2^ (15.7%) are freshwater bodies (lakes, ponds, rivers). Based on the CORINE Land Cover 2018 data, approximately 60% and 10% of the land area are forests and mires, respectively, 4% is urban and other built-up environments and 9% is in agricultural use (Fig. 2). The majority (71%) of the forests are coniferous (> 75% of trees conifers), while 4% and 24% are deciduous (> 75% deciduous trees) and mixed, respectively. The dominant tree species are Norway spruce (*Picea abies*), Scots pine (*Pinus sylvestri*s), silver birch (*Betula pendula*) and downy birch (*Betula pubescens*). Of the forests, 12% are grown on drained peatlands. Approximately 25 130 ha of the forests are clear-cut, and another 93 000 ha managed (e.g. thinned) every year in this region (Vaahtera et al. 2021). All data used in this study were scaled to a uniform 96 × 96 m resolution grid covering the entire study area but excluding water bodies.

### Forest and carbon data

We used PREBAS to simulate forest variables and carbon balance. PREBAS is a C-balance-based stand growth and gas exchange model, which combines a process-based forest growth model (called CROBAS, Valentine and Mäkelä 2005) and a daily canopy gas exchange model (PRELES, Peltoniemi et al. 2015). Photosynthesis (GPP) and evapotranspiration are calculated using a light-use efficiency approach linked to soil moisture and driven by daily climate information and ambient CO_2_ concentration. GPP is allocated to mean-tree growth and respiration at an annual time step. To calculate net ecosystem production (NEP), PREBAS has been linked through annual litter inputs with the soil C model YASSO15, which has been calibrated to one meter depth (Viskari et al. 2020). PREBAS has been calibrated with Nordic eddy covariance sites and Finnish growth experiments (Minunno et al. 2019).

PREBAS produces an array of outputs. Of these, we used the stand volume, age, dominant tree species, mean tree height and mean diameter at breast height (DBH) (Table 1) together with information on forest site type to represent the forest variables in our analysis. To represent carbon values, we used the PREBAS estimated amount of carbon stored in trees, ground vegetation and soil and the annual size of carbon sinks of the stand, calculated from NEP.

**Table 1:** Selected PREBAS outputs used in this study. The interlinked submodels of PREBAS simulate process-based forest growth (CROBAS), daily canopy gas exchange (PRELES) and soil carbon through annual litter inputs (YASSO). For references and further details of the submodels, see the main text

Variable name	Meaning	Unit	Submodel
DBH	Stand mean diameter at breast height	cm	CROBAS
Height	Stand mean height	m	CROBAS
Vol	Stand stem volume	m^3^ ha^−1^	CROBAS
Dec. vol	Deciduous stem volume	m^3^ ha^−1^	CROBAS
Age	Dominant tree age	years	CROBAS
Species	Dominant tree species	Pine, spruce, deciduous	CROBAS
Soil C	Total soil carbon	kg C ha^−1^	YASSO
Ground veg C	Total carbon in ground vegetation	kg C ha^−1^	Ground vegetation submodel (see Junttila et al. 2023)
Tree C	Total carbon in tree biomass	kg C ha^−1^	CROBAS
NEP/C sink	Net ecosystem production/size of carbon sinks	g C m^−2^ year^−1^	PRELES + CROBAS + YASSO

To initiate PREBAS simulations, we used information on the initial state and type of the forest from the Multi Source National Forest Inventory 12 (MS-NFI 12, 2014–2018) maps, which describe the forest parameters across Finland at 16 × 16 m resolution (Tomppo et al. 2008). To reduce computational effort, the simulation was done on homogeneous segments consisting of multiple 16 m pixels (Haakana et al. 2022). Within segments, the initial value and growth in forest were assumed the same for all pixels. Information on forest site type was extracted directly from the MS-NFI data. Due to the sparsity of measurement-based estimates, setting initial carbon content of mineral soils is challenging and was therefore assumed to be in a steady state with the historical mean levels of harvest (round wood, pulpwood and energy wood). After each simulation, the results were restored back to the 16 m resolution and aggregated to the 96 × 96 m analysis resolution (Forsius et al. 2021). For the conservation prioritisation, we only considered carbon sinks by setting NEP values to zero in pixels that on average acted as a source across the simulated time period. This is in line with current conservation policies where areas of large sinks may be favoured but areas of large sources are not penalised if the site is otherwise important for preserving biodiversity. For calculating regional carbon fluxes, both sinks and sources were considered.

Forest growth was simulated from 2015 to 2050 with a two-year initiation period (2015–2016). For transparency, during the simulations we assumed no climate change or further forest harvesting to take place, as our goal was to understand how input data uncertainty affects the spatial distribution of conservation priorities. The output variables from the simulations were produced as averages for three time periods: years 2017–2025 (T1, 9 years), 2026–2033 (T2, 8 years) and 2034–2050 (T3, 17 years).

### Biodiversity data

For biodiversity, we used the nesting suitability maps of six forest-dwelling bird species: European honey buzzard (*Pernis apivorus,* PERAPI), northern goshawk (*Accipiter gentilis,* ACCGEN), common buzzard (*Buteo buteo,* BUTBUT), white-backed woodpecker (*Dendrocopos leucotos*, DENLEU), lesser-spotted woodpecker (*Dryobates minor,* DRYMIN) and Eurasian three-toed woodpecker (*Picoides tridactylus*, PICTRI). The nesting sites of these species have been shown to be good indicators of forest biodiversity, as they prefer mature or undisturbed forest stands, mosaics of forest types and deciduous forests that have high dead wood volume and richness of wood-decaying polypore species (Roberge and Angelstam 2006; Burgas et al. 2014). All three hawk species and the white-backed woodpecker are red-listed (threatened or near-threatened, Hyvärinen et al. 2019) and the lesser-spotted woodpecker and three-toed woodpecker have shown considerable long-term declines in Finland (Väisänen et al. 1998).

The nesting suitability maps were produced using the species distribution modelling (SDM) tool Maxent (Phillips et al. 2006). As SDMs can account for a broader suite of variables (climate, non-forest habitats) and spatially relevant ecological drivers, such as the size and configuration of suitable forests and the quality of matrix (area between forests), they provide ecologically more realistic estimate of the goodness of forests for biodiversity than simple stand characteristics. We used bird ringing data independent from the MS-NFI to mark the locations of nesting sites of each species, and sets of environmental variables that describe the climate, forest and land cover characteristics at nesting site and landscape level (Table 2). Virkkala et al. (2022) modelled the nesting suitability of these species at the national scale and here we replicated these models using the same nesting records and species-specific sets of variables identified by Virkkala et al. (2022) (Table 2) but cut to the study area. The modelling was done using R v.4.1.0, Rstudio v.2022.07.01 and the package *dismo* v.1.3-5. We used the default settings of Maxent. Likely spatial biases in the ringing data were accounted for through the use of a bias grid and target group sampling (Phillips et al. 2009; Virkkala et al. 2022). The models were validated using a fivefold cross-validation to calculate the mean and standard deviation of the Area Under the receiver operating characteristic Curve (AUC), which is a commonly used metric to describe how well the model discriminates between known presences and absences. AUC values > 0.7 are thought to indicate an informative model (Fielding and Bell 1997) (Table 2). For the uncertainty analysis, we used models that were fitted using all available nesting records. We did not threshold the predicted values.

**Table 2:** Number of nesting records and the environmental variables used in the nesting suitability models for each bird species, and the average test AUC value and standard deviation of the models across fivefold cross-validation. For each variable, the values give the percent contribution and permutation importance (in brackets) of the full model using all records. The first value describes how much the variable contributes to the full model. The second value shows how much the model AUC is reduced when the variable is not included in the model. The three most important variables for each species in terms of contribution and permutation are in bold. LS refers to variables that were calculated at landscape level, either as averages (forest and climate variables) or proportion (land-use category), within a radius of either 500 m or 1 km around the nesting site, depending on the species (woodpeckers and hawks, respectively). Fixed variables are those that were kept constant in all model samples, whereas the others were replaced with a newly sampled data layer, see main text for details. *PERAPI* European honey buzzard, *ACCGEN* northern goshawk, *BUTBUT* common buzzard, *DENLEU* white-backed woodpecker, *DRYMIN* lesser-spotted woodpecker, *PICTRI* Eurasian three-toed woodpecker, *DBH* Diameter at breast height, *LS* Variable calculated at landscape level around the nesting site

		Hawks			Woodpeckers		
		ACCGEN	BUTBUT	PERAPI	DENLEU	DRYMIN	PICTRI
**Number of records**		928	356	75	64	27	56
**Test AUC**		0.795 ± 0.014	0.780 ± 0.024	0.726 ± 0.052	0.976 ± 0.003	0.835 ± 0.082	0.880 ± 0.063
**Environmental variable**	**Fixed**						
Stand volume (m^3^)		8.8** (43.1)**	**39 (18.7)**	**58.3 (48.3)**		0.0 (0.0)	0.0 (0.0)
Stand DBH (cm)		**22** (1.3)			2.7 (5.0)		
Stand height (m)			7.2 (13.2)	**10.8** (0.0)	4.7 (0.0)		**25.9** (4.6)
Dominant tree age (years)		**38.6 (18.9)**	7.7 (10.2)			1.9 (2.1)	1.4 (2.1)
Deciduous tree volume (m^3^)			4.1 (8.2)	3.3 (13.4)	**28.3 (37.6)**	5.0** (24.1)**	
Main tree species				**16.5 (14.5)**	1.6 (0.8)	1.5 (0.0)	6.7 (2.7)
Forest site type		3.2 (2.2)		1.4 (3.4)	4.6 (0.1)		3.0 (1.4)
Shoreline forest (%)	x					11.7 (5.3)	
Agricultural areas (%)	x	1.4 (6.4)					2.3 (6.9)
Urban areas (%)	x	1.2 (0.1)	4.8 (1.0)	4.5 (5.0)			
Water areas (%)	x	3.1 (6.7)	1.9 (3.5)				
LS volume at 500 m radius (m^3^)					1.4 (2.5)		**21** (1.0)
LS volume at 1 km radius (m^3^)		0.2 (0.3)	2.6 (4.0)				
LS site type at 500 m radius (%)					5.3 (0.1)	**16.2** (4.6)	11.8** (17)**
LS site type at 1 km radius (%)			1.9 (2.7)	0.6 (0.4)			
LS shoreline forest at 500 m radius (%)	x				**17.1** (4.7)	**20.9 (36.6)**	
LS forest on peatland at 500 m radius (%)	x				7.1** (30.9)**	0.4 (1.8)	
LS marshlands at 500 m radius (%)	x				2.5 (0.0)	3.6 (2.0)	
LS agricultural areas at 1 km radius (%)	x		2.2 (2.0)	4.5** (15.0)**			
LS urban areas at 500 m radius (%)	x					**34.2 (17.9)**	8.1 (8.4)
LS urban areas at 1 km radius (%)	x	**17 (18)**	**7.8 (18.6)**				
LS water areas at 500 m radius (%)	x				2.4 (0.2)	0.9 (5.7)	3.9** (19)**
LS water areas at 1 km radius (%)	x		**18.9 (13.8)**				
January mean temp (°C)	x	4.4 (2.9)			**22.4 (18.1)**	3.7 (0.0)	**15.9 (36.8)**
GDD5	x		1.8 (4.2)	0.0 (0.0)			

### Sample iterations

The baseline forest variables were simulated using existing forest data (MS-NFI). To create sample forest variables, we iteratively repeated the PREBAS simulation while introducing three sources of uncertainty: (1) variable input uncertainty; (2) model parameter uncertainty and (3) uncertainty in weather conditions (Table S1).

To introduce variable input uncertainty, the initial segment-level variables (structural, site type and age) were resampled from their respective estimate distributions. For structural variable values (basal area split to proportions of pine, spruce and birch, stand mean height and mean DBH), estimate distributions were produced using data from three remotely sensed 100 × 100 km tiles across Central Finland from Miettinen et al. (2021) and assuming multivariate normal distribution. Variables were resampled simultaneously for each segment using a covariance matrix of errors. To avoid negative or unrealistically large values, and to maintain the covariance structure between variables, the segment samples were post-processed using the quantile matching procedure (Junttila and Kauranne 2018). For site type, data on the satellite-based site type and forest structural variables from Miettinen et al. (2021) were used together with a probit model to calculate the probability of a segment belonging to each of the site type classes. Those probabilities were then used to resample the forest site type at the start of each iteration to reflect potential uncertainty in the initial classification of the MS-NFI. Uncertainty in the segment mean age was estimated using random samples from a normal distribution around MS-NFI-based mean age (across all tree species) and 10% standard deviation.

As the MS-NFI data does not include information about height of the crown base, we used species-specific empirical models fitted to data from permanent sample plots (Minunno et al. 2019) and varied the estimates with 10% standard deviation (estimate multiplied with *h* ~ *N*(1, 0.1^2^)).Otherwise, model parameter uncertainty was generated by bootstrapping the joint posterior distributions of PRELES, CROBAS and YASSO parameters from Minunno et al. (2016), Minunno et al. (2019) and Viskari et al. (2020). The sampling strategy respected parameter correlations and interactions. Weather condition uncertainty was simulated by sampling yearly weather conditions from the last 46 years (1971–2016) to represent the local weather during the simulation period.

In total, we ran 50 iterations of the PREBAS simulation. In each iteration, the values for site type and PREBAS parameters were sampled at the start and then kept constant across the simulation time period. For more details on the uncertainty sampling, see Junttila et al. (2023).

### Spatial prioritisation

Priority areas for protection were identified using the spatial prioritisation software Zonation v.5.0 (Moilanen et al. 2009; Moilanen et al. 2022). Zonation produces a priority ranking of each spatial unit (here 96 × 96 m grid cells) based on input feature data (6 bird species and 4 carbon layers), ordering the units from the least to the most important for conservation. This is done in an iterative optimisation algorithm where the software seeks to find a rank order that maximises the representation of all features in the top ranked grid cells. Consequently, a set of top ranked priority areas together typically capture high value areas for all input features. For each feature *j*, we used a benefit function *v*_*j*_ (*r*_*j*_) = *r*_*j*_^*z*^ to describe the value of added protection *v*_*j*_ as a function of the accumulating fraction *r*_*j*_ of feature *j*’s full distribution that is protected. The parameter *z* defines the shape of the function. We set *z* = 0.25 for all biodiversity features (bird species), following the well-established species-area relationship (Rosenzweig 1995). For carbon features, we used *z* = 1 as, unlike species persistence, the persistence of carbon in the landscape does not have a non-linear relationship with the remaining area.

During the prioritisation, feature-specific benefits *v*_*j*_ of protecting a grid cell *i* were treated as additive. We produced a priority ranking for the baseline and each sampled iteration of the biodiversity and carbon features, and selected the top ranked 10% of the grid cells to represent the potential conservation solution for that iteration.

### Quantification of uncertainty

We quantified uncertainty by measuring across iteration variability in (i) the PREBAS simulated forest variable and carbon values per hectare, (ii) amount of suitable bird habitat in the region and (iii) within grid-cell priority ranking and the frequency at which any one grid cell was included in the top 10% ranking. Although some of the forest variables correlate (volume, height, DBH), we examined them all as they have varying importance for dependent biodiversity (Table 2).

For each sample iteration and time period, we extracted forest variable, biodiversity, carbon and conservation priority values from 10 000 grid cells across the study region. We conducted a canonical correlation analysis (CCA), a multivariate extension of correlation analysis that allows identifying linear relationships between two datasets consisting of one or multiple variables (Stewart and Love 1968). We summarise the CCA results with the use of the redundancy index that expresses the amount of variance in the within-pixel priority rankings explained by each variable (van den Wollenberg 1977). The index receives values 0–1, where higher value indicates that the variable explains more of the variation, but there is no agreed interpretation of the exact value. Therefore, we examine the derived redundancy indices of each variable in relation to one another to reveal relative uncertainties. To better understand the explanatory power of the variables, we included three prioritisations in the CCA which were based on (i) both biodiversity and carbon features, (ii) biodiversity only and (iii) carbon only. We summarise the result for only (i) in the main text, see the Supplementary for all results.

## Results

### Changes and uncertainty input data estimates

**Fig. 3:**
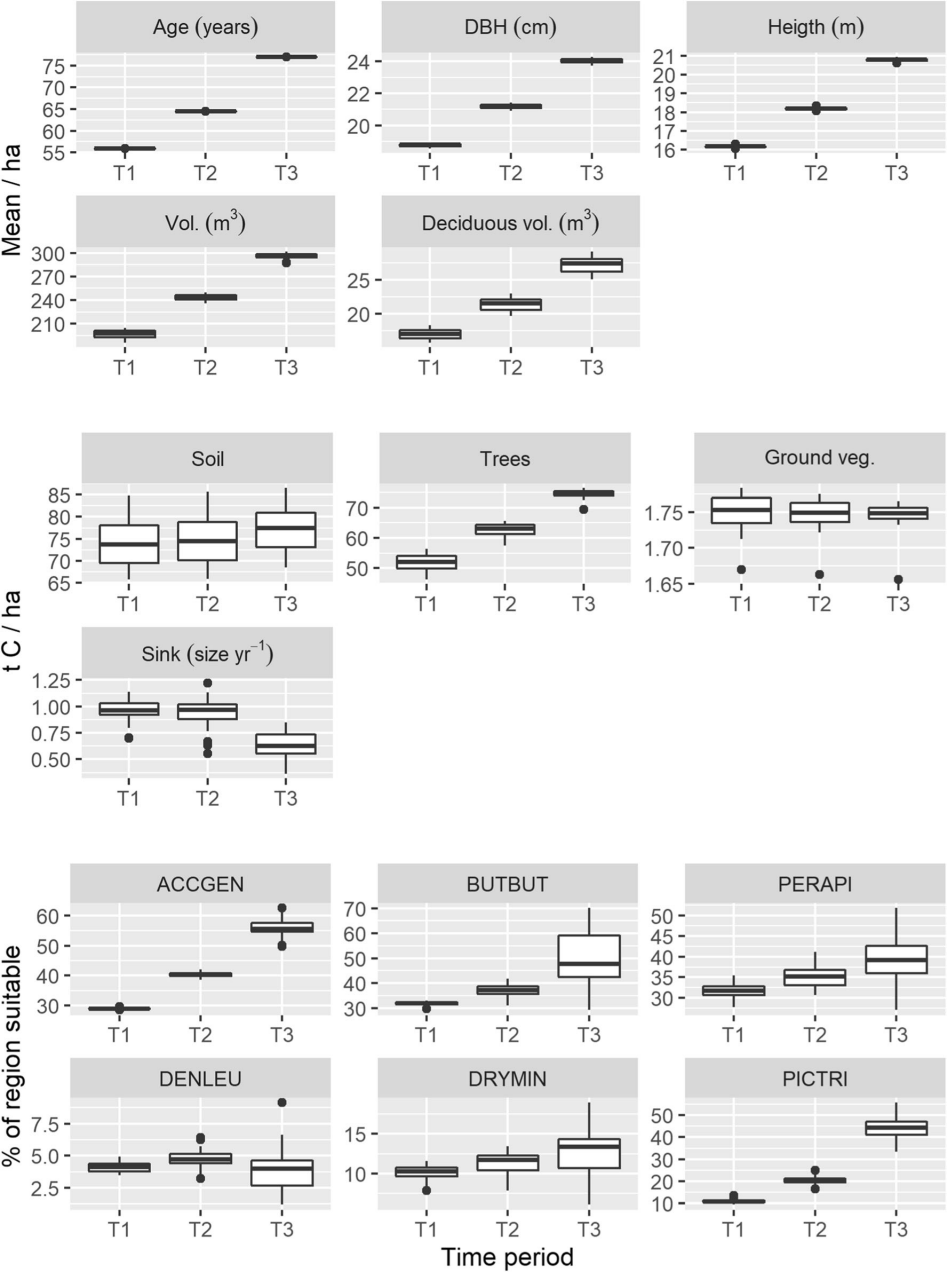
Variation in the sampled forest variables and the respective carbon and biodiversity values. For the forest variables and carbon features, the values are given as means across study region. For segment-level means and variation, see Table S2. For the biodiversity features, the proportion of study area that is suitable is given, calculated as the sum of predicted values divided by the study area. *DBH* tree diameter at breast height, *ACCGEN* northern goshawk, *BUTBUT* common buzzard, *PERAPI* European honey buzzard, *DENLEU* white-backed woodpecker, *DRYMIN* lesser-spotted woodpecker, *PICTRI* Eurasian three-toed woodpecker. Note that the y-axes are not fixed but are on different scales

In the absence of harvesting, the tree height, DBH, volume and age of forests increased (Fig. 3). There was little variation in the across study area mean of forest variables between sample iterations (for segment-level variation, see Table S2). The volume and proportion of deciduous trees also increased (Fig. 3, from 8.6 at T1 to 9.2% at T3), mainly due to intraspecific competition and the domination of deciduous tree species during the re-growth of recently clear-cut and early succession forests that were initially present in the region.

The amount of carbon stored in trees and soil increased from 473 to 571 million t C (+ 20%) across the region (Fig. 3), as no carbon was removed from the system through logging. Uncertainty around the tree carbon estimates decreased towards the last time period, whereas the estimates of soil carbon remained more variable. In contrast, ground vegetation carbon stayed relatively stable and became less variable through time. The total size of carbon sinks decreased across the region, from 3.6 to 2.3 million t C year^−1^ (− 36%), as tree growth slowed down in the maturing forests. Uncertainty around carbon sinks remained stable across time. There was more variability around the carbon estimates in comparison to forest variables (Fig. 3).

The undisturbed maturation of forest stands led into large increases in the amount of suitable nesting habitat for most bird species, in particular for the northern goshawk, common buzzard, the three-toed woodpecker but also the European honey buzzard (Fig. 3). Wood volume, tree height, DBH and age of forest stands were some of the strongest predictive variables for these species (Tables 2, S1) and all these increased through time. Although deciduous tree volume was a major predictor for the lesser-spotted and white-backed woodpeckers (Table 2), its increase did not translate into notable improvements in their nesting habitat (Fig. 3). This might be because: (i) the additional deciduous trees were mixed with conifers and these species prefer pure deciduous forests, (ii) the habitat increase of the lesser-spotted woodpecker is constrained to nearby water bodies and (iii) in the absence of disturbances, such as fires, old-growth deciduous forests preferred by the white-backed woodpecker tend to become overgrown by conifers (Fig. S2, Tables 2, S1). For all species, the estimated amount of available habitat became more uncertain through time, being largest for the common buzzard (Figs. 3, S1). There was no clear relationship between model fit (test AUC values, Table 2) and the variation in the amount of available habitat (Figs. 3, S9).

### Changes and uncertainty in conservation priority rankings

**Fig. 4:**
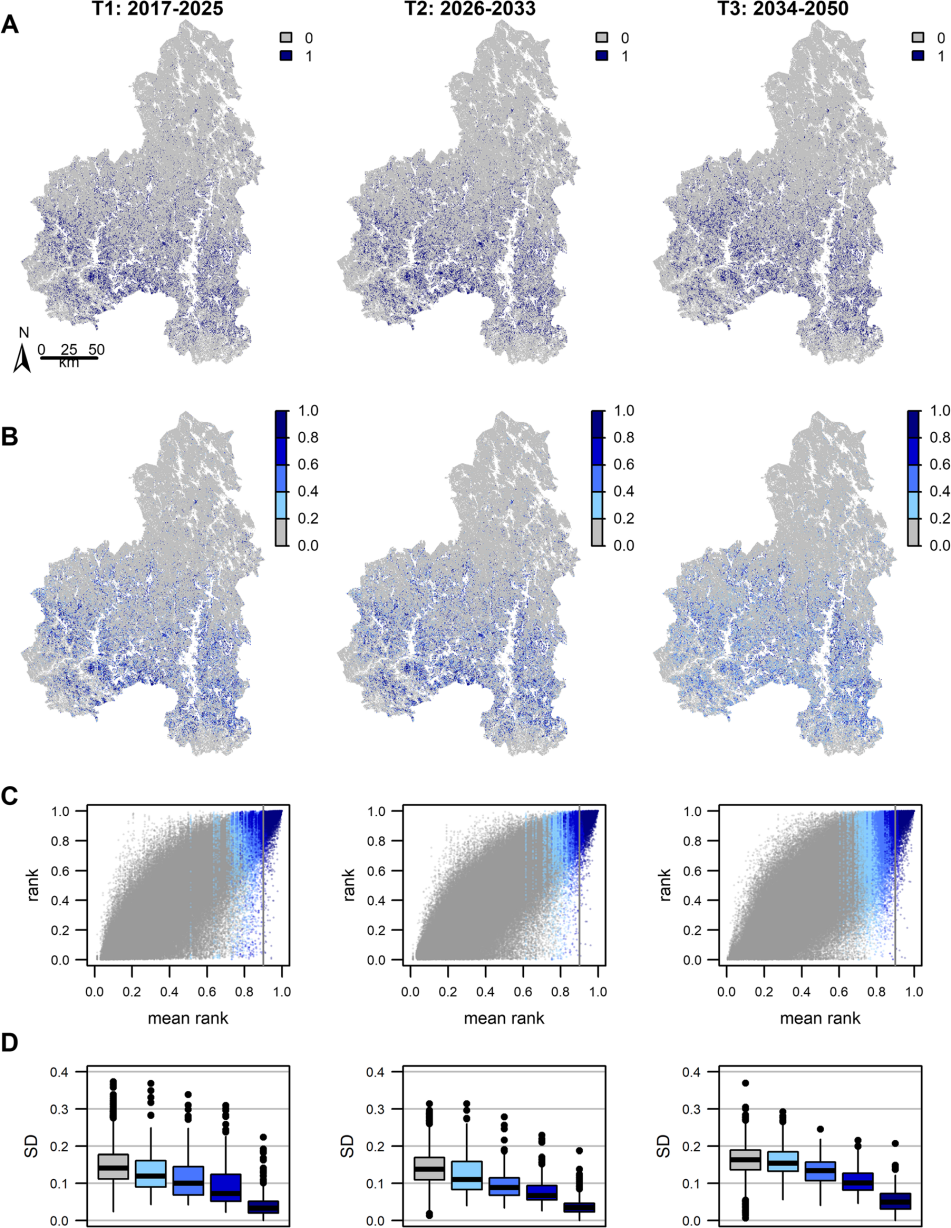
Priority areas for conservation and their associated uncertainty in each time period. For each time period, a baseline and 50 sample prioritisations were produced using the resampled forest variables and their corresponding carbon and bird distribution maps. Panel **A** shows the top ranked 10% of grid cells (dark blue) for baseline data at each time period. Panel **B** gives the probability of a grid cell to be in the top 10% across the 50 sample prioritisations. The scatterplots (**C**) show the mean (*x*-axis) and range (*y*-axis) in rank values for each grid cell across the sample solutions. Here grid cells have been ordered based on their mean rank values from lowest (left) to highest (right) priority and for each cell the points give the rank value for one iteration. For visibility, these are shown for a subset of 4000 grid cells. The point colours correspond to the probability of the cell to be within the top 10% across samples, as in **B**. The boxplots in panel **D** show the distribution of standard deviations (SD) in the rank values for grid cells. The colours correspond to each of the probability groups shown in **B** and **C**

Across the time periods, the priority rankings of forests for biodiversity and carbon increased in the southeast of the region, while forests in the northern parts of the region became less important (Fig. 4). By the last time period, there were only few forests ranked in the top 10% in the northern parts, while their locations shifted and became more evenly spread in the south-west (Figs. 4A, S4).

The ranking of individual grid cells varied notably across the sample iterations in each time period (Fig. 4C, vertical spread of points). This variation was reduced in the second time period, but increased again in the third time period (Figs. 4D, S5). There were no strong spatial patterns in the variability of cell rankings (Fig. S5). In all cases, grid cells that were on average ranked as the highest or the lowest priority across the 50 iterations showed least variation (Fig. 4C, D).

Still, even for the on average top 10% ranked grid cells (points right from the vertical line in Fig. 4C), this variation was large, and in some iterations, these cells were ranked as moderate or low priority. Furthermore, by the third time period, variation in the cell rankings increased more in the high ranked than low ranked cells (Fig. 4C, D). From this followed that the probability of any one grid cell to be included in the top 10% in all iterations decreased (Fig. 4B) and a larger number of grid cells could be ranked as top priority in at least one of the iterations (Fig. 4C, area of coloured points).

### CCA

**Fig. 5:**
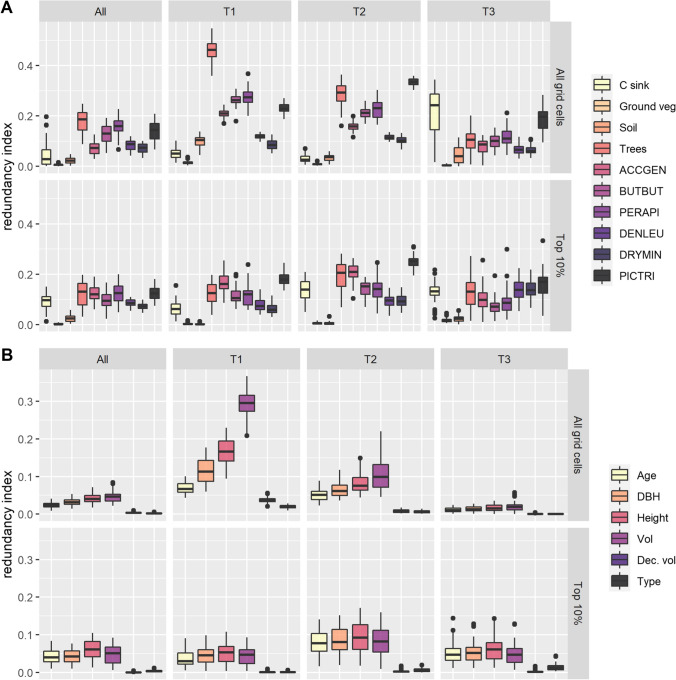
Redundancy indices of different modelled input variables from the canonical correlation analysis. Higher values of the redundancy index mean the feature explains more of the variation in the priority rankings. The boxplots show how different input features (A. carbon and biodiversity, B. forest variables) explain the variance in the priority rankings of all grid cells (upper row) and in the top 10% ranked grid cells (lower row) and how much this varies across the 50 sample iterations (boxplot). The results are broken down for each time period (T1, T2, T3). The leftmost panel (“All”) shows the canonical correlation across all time periods. The redundancy index values are comparable between columns, but not between rows, as these measure correlations between different sets of input features and priority solutions (see text). *Ground veg* ground vegetation carbon, *ACCGEN* northern goshawk, *BUTBUT* common buzzard, *PERAPI* European honey buzzard, *DENLEU* white-backed woodpecker, *DRYMIN* lesser-spotted woodpecker, *PICTRI* Eurasian three-toed woodpecker, *DBH* tree diameter at breast height, *Dec. vol* volume of deciduous trees, *Type* forest site type (fertility). Note that the scales of the *y*-axis differ across panels

Across all grid cells, the variance in priority ranking was most strongly and consistently explained by the amount of carbon stored in trees and the distribution patterns of three-toed woodpecker, honey buzzard and common buzzard (Fig. 5A, upper row). Of the initial forest variables, tree volume followed by tree height and DBH explained most of the variation seen in priority rankings of all grid cells, particularly in the first two time periods (Fig. 5B). Tree volume and tree carbon storage correlate closely, in addition to which tree volume is one of the strongest predictors of the nesting suitability for several of the bird species (Table 1). Hence, minor changes in tree volume, and thereby tree carbon storage, may trickle into the prioritisation results via multiple input features, even if carbon features are not included in the prioritisation (Fig. S7).

By the third time period, most of the forest stands had become similar in terms of biomass and structural variables (Figs. 3, S3), and the forest variables alone had little effect on how grid cells were ranked. Concurrently, the importance of the tree carbon storage, common buzzard and honey buzzard on the ranking were reduced, while the importance of the three-toed woodpecker remained high (Fig. 5A, upper row). In the last time period, changes in the carbon sink estimates greatly affected the priority rankings, although this effect in itself was highly variable across the 50 iterations.

In contrast to all grid cells, variation in the top 10% ranked cells was more evenly explained by both carbon and biodiversity features (Fig. 5A, lower row). This is logical as the prioritisation seeks to rank highest those areas that capture the high-value locations of all input features. Variation was predominantly explained by the three-toed woodpecker, northern goshawk and tree carbon storage on the first time period and became balanced between tree carbon storage and sinks and the three woodpecker species in the last time period. Unlike in the full ranking, the variation in the top ranked 10% grid cells was consistently explained most by the forest structural variables describing the mean height, volume and DBH together with dominant tree age of the forest stands, although the explanatory power was low.

The CCA patterns were consistently similar for prioritisation based only on biodiversity, whereas in the carbon-only prioritisation uncertainty in the ranks were predominantly only explained by carbon sinks (Figs. S7, S8).

## Discussion

Our results illustrate the complex nonlinearities between model input and output uncertainties across the different modelling levels (forest variables, carbon and biodiversity features, priority rankings). The seemingly small alterations in the simulated forest variables often resulted in much larger variation in carbon and biodiversity estimates (Fig. 3). To some degree, these are explained by the different ways uncertainty accumulates in these estimates. For example, the large variation around soil carbon is likely driven by the uncertainty in the initial estimate (not tested here), which assumes steady state and is in turn affected by uncertainty around PREBAS parameters and forest management (Junttila et al. 2023). Variation around biomass and carbon stored in trees and ground vegetation mostly stems from the input variable uncertainty, that is, variation in the initial structural forest variables and mean age, which becomes reduced as the simulated forest growth evens out differences between forest stands (Junttila et al. 2023). In contrast, uncertainty around the carbon sink estimate accumulates from multiple submodels of PREBAS (Table 1). Unlike input variable uncertainty, the effect of PREBAS parameter uncertainty increases with time as model outputs generated with different parameter vectors become more diverged (Mäkelä et al. 2020; Junttila et al. 2023), increasing uncertainty in the sink estimate. Similarly, despite the perceived certainty around forest variable estimates, the bird habitat estimates become more uncertain with time (Fig. 3) as the combined environmental conditions (forest and non-forest variables) of the region becoming increasingly different from those under which the model was calibrated (Barry and Elith 2006).

On the other hand, the priority rankings of grid cells showed both spatial stability (Figs. 4B, S5) and disproportionally large within-pixel variability (Fig. 4C) in comparison to uncertainty around carbon and biodiversity estimates (Fig. 3). The latter is explained by the way values within each biodiversity and carbon feature are distributed (Fig. S6). For all features, the highest values tend to be rare. Most of the grid cells, therefore, have low (biodiversity) or intermediate (carbon) values and their relative conservation importance becomes similar. Hence, even modest alterations in the raw carbon and biodiversity values can drastically change the priority of these middle-low ranked cells (Kujala et al. 2018b). In contrast, the few grid cells with the highest biodiversity and carbon values constrain the top rankings to these locations. As the sample iterations do not significantly alter the shape of the value distribution, the top ranked grid cells remain more stable. Finally, the study area has some 3.8 million grid cells: even very large variations in single pixel rankings (Fig. 4C) do not necessarily translate into changes in the overall spatial patterns (Fig. S5). These observations show that the connections between input data uncertainty (forest attributes, carbon and biodiversity patterns) and decision uncertainty (priority rankings) are complex, and that care must be taken in interpreting how one influences or is driven by the other.

The need to reduce uncertainty around input data depends on how it affects the decision being made (Howard 1966). Refining estimates of those input data that explain most of the variation in priority ranks is the most logical starting point, and in our study case, the uncertainty around placement of protected areas (top 10% ranked sites) would be most reduced by improving our understanding on the distribution of the three-toed woodpecker, the northern goshawk, tree carbon storage and carbon sinks (Fig. 5). A more detailed uncertainty analysis of the different modelling approaches, such as done by Junttila et al. (2023) for PREBAS, can reveal strategies for how to best achieve this. However, our results reveal also other important observations. First, which input data introduces most of the uncertainty in the priority solution depends on what fraction of the landscape we focus on (Fig. 5). Second, high variability in predicted values or differences in model fit (AUC, Table 2) indicated poorly which input feature introduced most uncertainty in the priority ranking (Fig. S9). Instead, the spatial rarity, nestedness and co-occurrence with other input features explains how much of the ranking is driven by an input variable (Kujala et al. 2018b). For example, as tree carbon storage, common buzzard and honey buzzard became spatially more common with time (Figs. 3, S1, S3), there were more equally good options to protect them and they explained less of the ranking variability (Fig. 5a). The habitat of the three-toed woodpecker also increased (Fig. 3) but remained spatially confined (Fig. S2) and retained its high influence on the ranks. In contrast, the decrease in carbon sinks (Figs. 3, S3) increased its influence on the priority ranks. Carbon sinks also tend to have different spatial patterns in comparison to stored carbon and biodiversity (Fig. S6), as NEP is highest in relatively young, fast growing forest stands (Minunno et al. 2019). Since the spatial prioritisation aims to capture high-value areas of all input features, one feature with a strongly dissimilar distribution from others can influence the ranking more (Kujala et al. 2018b). This effect varied greatly between sample iterations (Fig. 5a), even though the increase in uncertainty around carbon sinks was only moderate.

Our work focused solely on input variable uncertainty. Most notably, we did not account for uncertainty arising from forest management actions and future climate change, which can have an even larger impact on forest and biodiversity estimates (Buisson et al. 2010; Mäkelä et al. 2020). Although this was necessary to single out the impacts of target uncertainties, real-world conservation planning would rarely happen in isolation from these threats, and our results particularly for future time steps need to be interpreted with this in mind. However, observing the changes in forests in the absence of harvesting allowed us to better understand why certain forest, carbon and biodiversity values become such important drivers of the spatial prioritisation solution.

Our results also need to be interpreted in light of the made modelling choices and their potential limitations. Although the area on which the original bird species distribution models (SDMs, Virkkala et al. 2022) were built overlaps with ours, and thus, the risk of model extrapolating is small, the transferability of these types of models can be poor (Barry and Elith 2006). We also focused on just one region and used one type of forest model, SDM method and spatial optimisation. The benefit of the chosen spatial prioritisation algorithm is that it does not require pre-defined conservation targets (such as percentage of area protected) to be set for each feature, which would add an arbitrary constraint and reduce the transparency and interpretability of the results. Nevertheless, there are many other options for modelling tree growth, carbon fluxes and species distribution patterns, and the selection of modelling method is a known source of uncertainty in itself (Buisson et al. 2010; Mäkelä et al. 2020). Including all these factors would likely result in higher uncertainty in the priority areas and carbon and biodiversity estimates than shown here.

## Conclusion

Input data uncertainty may lead to misguided spatial plans and decisions, resulting in excessive spending of resources or the neglect of targeted values in areas mistaken as unimportant. Although the conservation priority of individual grid cells was highly sensitive to input data uncertainty, the spatial patterns remained rather stable. In particular, the top priority areas were least sensitive, giving some confidence that spatial prioritisation-based conservation plans can be robust against data uncertainty.

We did not find evidence that forest conservation strategies would be more sensitive to uncertainties in either biodiversity or carbon data. The amount of uncertainty in feature estimates themselves, whether measured by variability in predicted values or model fit (AUC), was a poor indicator of which the input features introduced most variation in the priority rankings. Rather, their impact was explained by the combination of what fraction of the forests was being considered and the spatial rarity and co-occurrence of the input features. Thus, even small estimate variation in a very influential input feature (e.g. tree carbon storage or three-toed woodpecker at time period T1) could have large effect on the location of priority areas and vice versa (e.g. common buzzard at T3).

The presented approach allows identifying input features that most strongly define the conservation priority of forest stands, and which are less important to account for. This helps us to understand the relative importance of data uncertainties and how to most effectively reduce it when improving the accuracy of spatial prioritisation-based conservation plans.

### Supplementary Information

Below is the link to the electronic supplementary material.

Supplementary file1 (PDF 3497 KB)
