# Supplementary material for: Role of data uncertainty when identifying important areas for biodiversity and carbon in boreal forests
Source: Ambio. 2023 Sep 1;52(11):1804–18. doi: 10.1007/s13280-023-01908-2 (PMC10562324; doi:10.1007/s13280-023-01908-2)
Supplement: Supplementary file 1 — Supplementary file1 (PDF 3497 KB) [file 13280_2023_1908_MOESM1_ESM.pdf]

Ambio

Electronic Supplementary Material

Title: **Role of data uncertainty when identifying important areas for biodiversity and carbon in boreal forests**

Authors: Heini Kujala, Francesco Minunno, Virpi Junttila, Ninni Mikkonen, Annikki Mäkelä, Raimo Virkkala, Anu Akujärvi, Niko Leikola, Risto K. Heikkinen

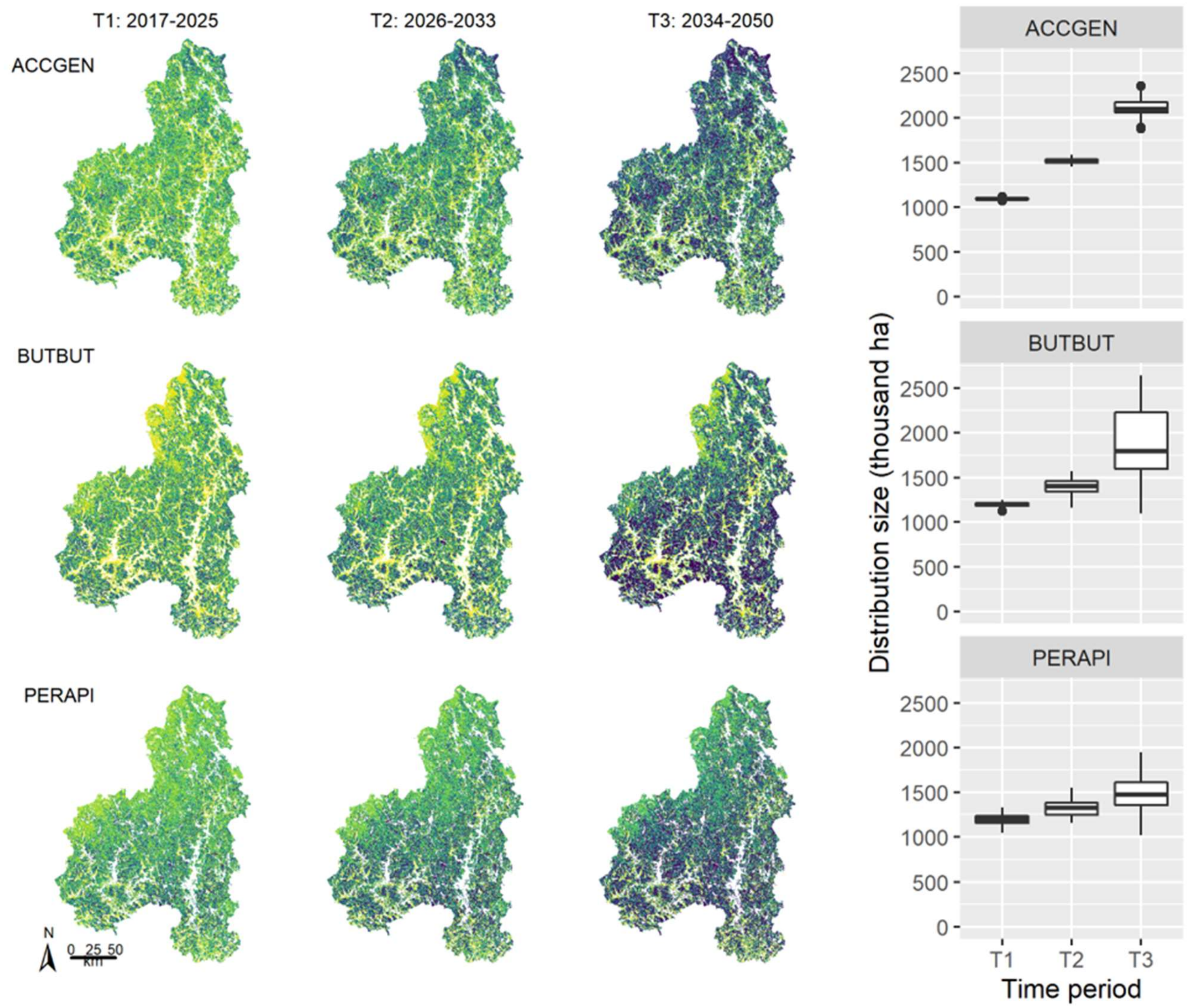

Figure S1. Predicted habitat suitability for the three hawk species at each time-period. Maps show the baseline habitat suitability. Darker color = higher suitability. Boxplots give the variation in the distribution size across samples and time-periods. ACCGEN = northern goshawk (*Accipiter gentilis*), BUTBUT = common buzzard (*Buteo buteo*), PERAPI = European honey buzzard (*Pernis apivorus*).

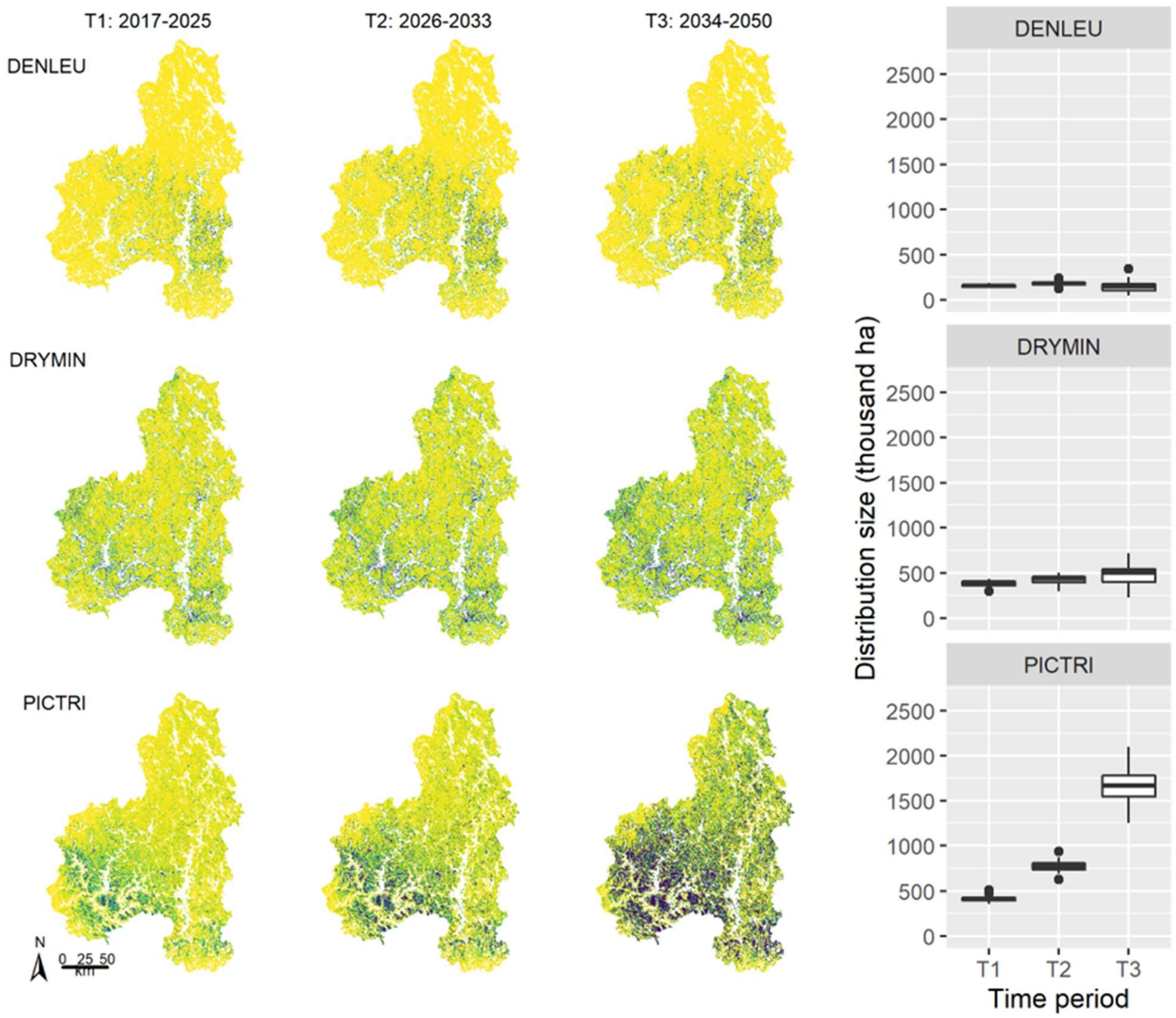

Figure S2. Predicted habitat suitability for the three woodpecker species at each time-period. Maps show the baseline habitat suitability. Darker color = higher suitability. Boxplots give the variation in the estimated distribution size across samples and time-periods. DENLEU = white-backed woodpecker (*Dendrocopos leucotos*), DRYMIN = lesser-spotted woodpecker (*Dryobates minor*), PICTRI = Eurasian three-toed woodpecker (*Picoides tridactylus*).

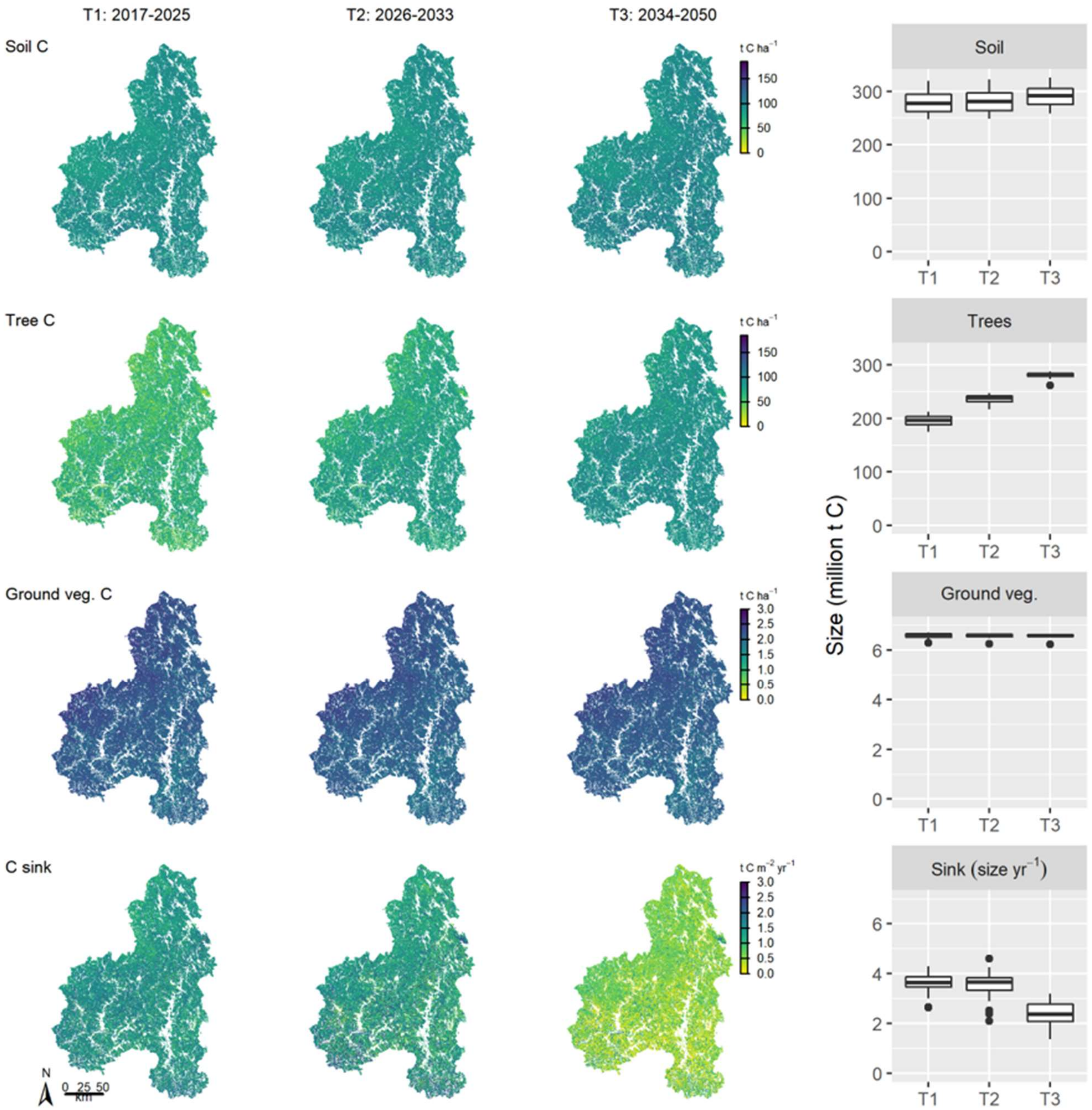

Figure S3. Predicted carbon values at each time-period. Maps show carbon values for the baseline models. Boxplots give the variation in the estimated total carbon storage and sink size for the region across samples and time-periods. C = carbon, Ground veg. = ground vegetation. Note that the boxplots are on different scales.

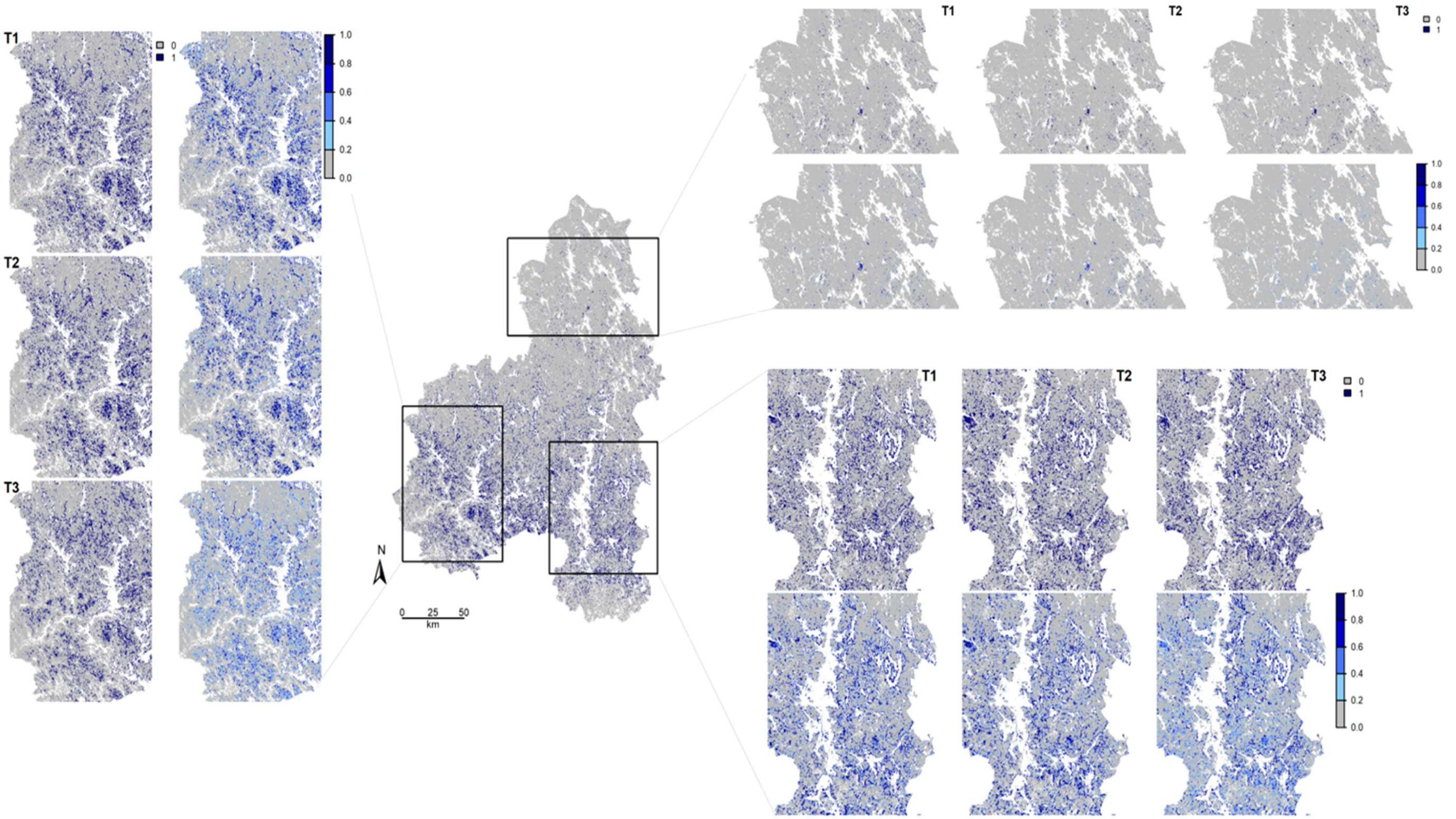

Figure S4. Spatial shifts in the top 10% ranked grid cells in different parts of the study region. The full map in the middle shows distribution of the top 10% ranked grid cells (in dark blue) at the first time period. The insets show in binary maps how the location of these priority areas change across time-periods (T1, T2, T3). The maps with gradient scale show the probably that a grid cell is ranked in the top 10% in each of the 50 sample iterations (see main text).

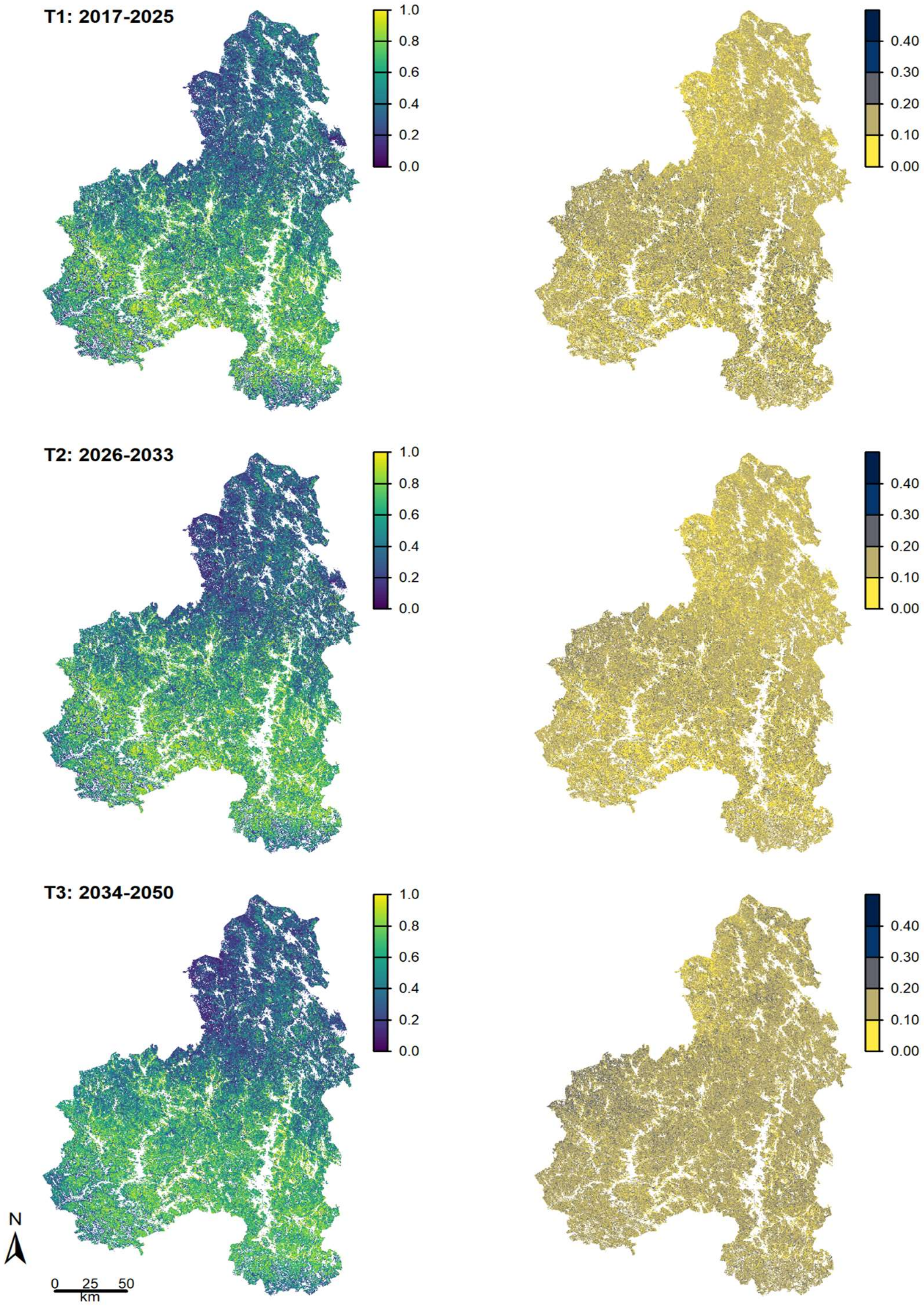

Figure S5. Mean priority ranking and standard deviation across sampled realisations. Each row corresponds to one time-period. The leftmost maps give the mean ranking for each grid cell across 50 priority rankings, light colors indicating high rank values and dark colors low. Standard deviations for each grid cell are shown in the rightmost maps (dark color = high SD).

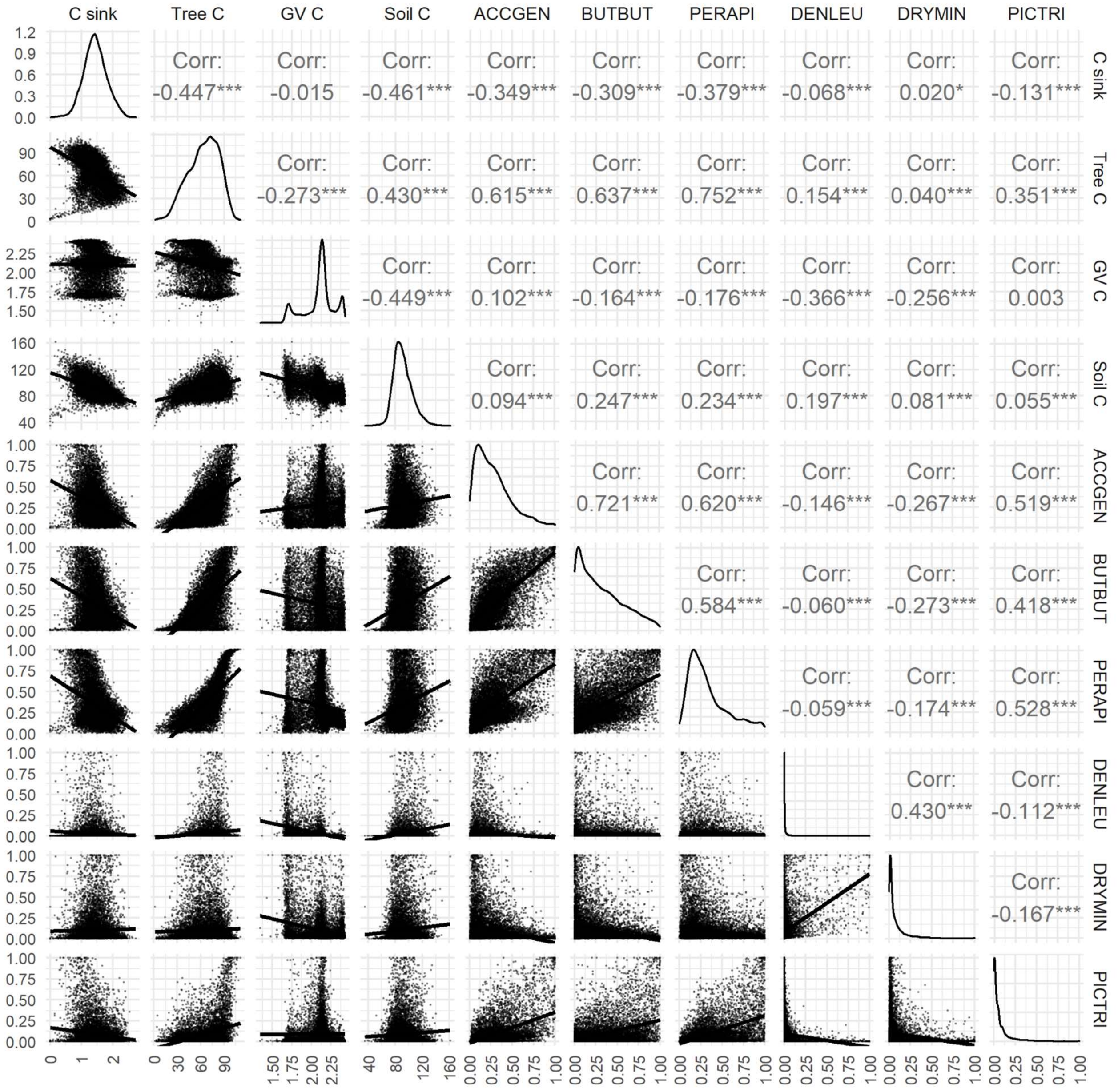

Figure S6. Correlation between carbon and biodiversity features in the baseline models for the first time-period (T1). GV = ground vegetation, ACCGEN = northern goshawk, BUTBUT = common buzzard, PERAPI = European honey buzzard, DENLEU = white-backed woodpecker, DRYMIN = lesser-spotted woodpecker, PICTRI = Eurasian three-toed woodpecker. Units for carbon sink and carbon stored in trees/ground vegetation/soil are g C m-2 yr-1 and kg C ha-1, respectively. For the bird species, values represent habitat suitability, ranging from 0 (low suitability) to 1 (high suitability).

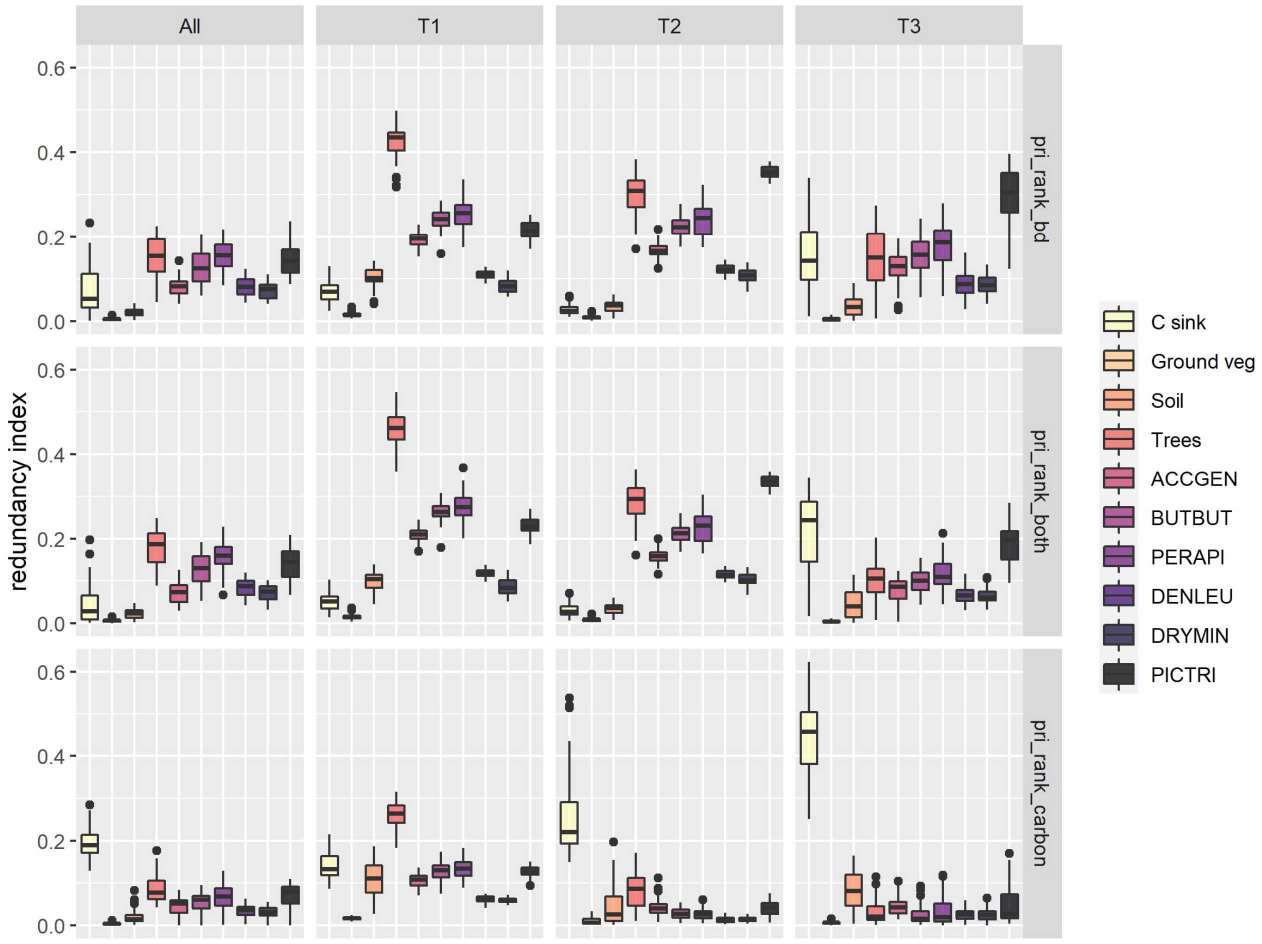

Figure S7. Results of the canonical correlation analysis showing how different input features (carbon and biodiversity) explain the variance in the priority rankings of all grid cells, and how much this varies across sample iterations (boxplots). The results are broken down for each time-period (T1, T2, T3) and prioritisation type (biodiversity only, biodiversity and carbon, carbon only). The leftmost panel shows CCA across all time-periods for each prioritisation type. Ground veg = ground vegetation carbon, ACCGEN = northern goshawk, BUTBUT = common buzzard, PERAPI = European honey buzzard, DENLEU = white-backed woodpecker, DRYMIN = lesser-spotted woodpecker, PICTRI = Eurasian three-toed woodpecker, DBH = tree diameter at breast height, Dec. vol = volume of deciduous trees, Type = forest site type (fertility).

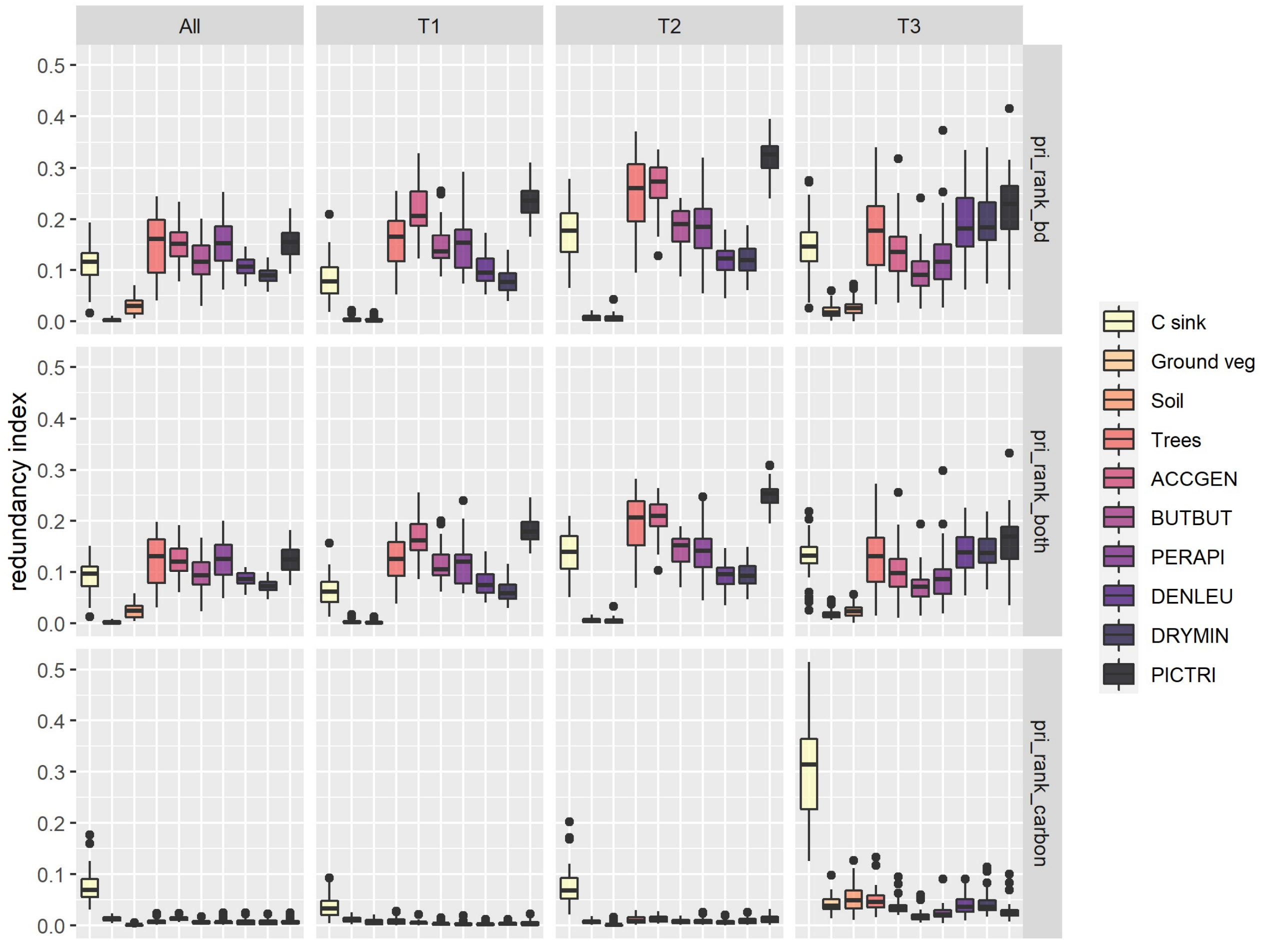

Figure S8. Results of the canonical correlation analysis showing how different input values (carbon and biodiversity) explain the variance in the priority rankings in the top 10% grid cells, and how much this varies across sample iterations (boxplots). The results are broken down for each time-period (T1, T2, T3) and prioritisation type (biodiversity only, biodiversity and carbon, carbon only). The leftmost panel shows correlation across all time-periods for each prioritisation type. Ground veg = ground vegetation carbon, ACCGEN = northern goshawk, BUTBUT = common buzzard, PERAPI = European honey buzzard, DENLEU = white-backed woodpecker, DRYMIN = lesser-spotted woodpecker, PICTRI = Eurasian three-toed woodpecker, DBH = tree diameter at breast height, Dec. vol = volume of deciduous trees, Type = forest site type (fertility).

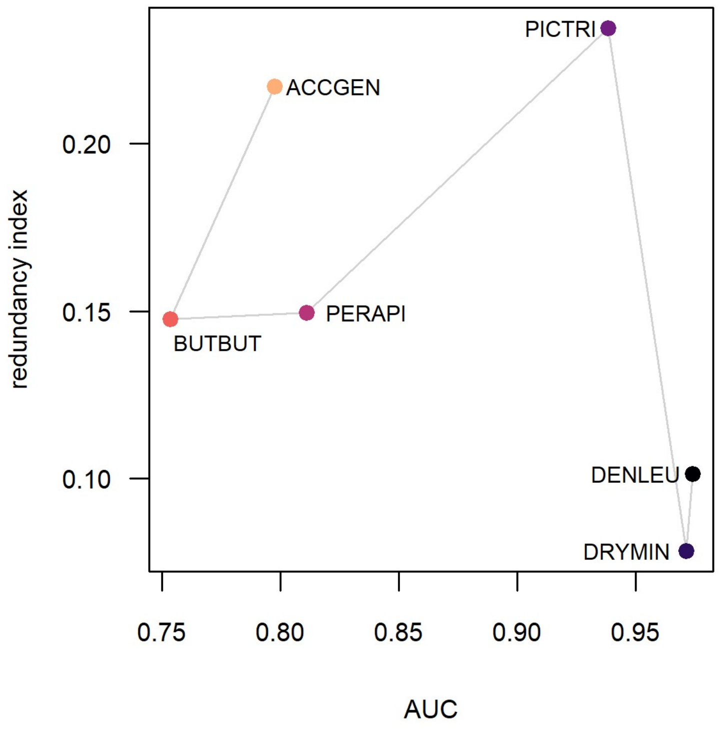

Figure S9. Relationship between model performance, estimate variation and redundancy index for top 10% ranked grid cells, shown for the six modelled bird species at the first time-period (2017-2025). The x-axis gives the mean predictive performance across 5-fold cross-validations, as measured by AUC. The y-axis shows the mean redundancy index across 50 sample iterations, indicating how much that species on average explains the variance in the priority rankings of the top 10% grid cells. The colors and the connecting line indicate increasing variation in the estimated distribution size, measured as coefficient of variance (CV) across the 50 sample iterations, ranging from lowest (light color, ACCGEN) to highest (dark color, DENLEU).

Table S1. The different sources of uncertainty used in the study to create sample forest variables. For full description of the uncertainty sampling and their effects in the PREBAS simulations, see Junttila et al. (2023).

Type of uncertainty	**Source**
Segment-level variable input uncertainty	Tree species-specific basal area
	Mean height
	Mean DBH
	Site type
	Initial age
Parameter uncertainty	Crown height factor
	PRELES parameters
	CROBAS parameters
	YASSO parameters
Weather	Weather

Table S2. Segment level uncertainty of forest and carbon variables. The table gives the mean and standard deviation (SD) of variable values across segments and their coefficient of variance (CV) across the 50 sample iterations and for each time period.

Variable	***T1***		***T2***		***T3***	
	**Mean ± SD**	**CV**	**Mean ± SD**	**CV**	**Mean ± SD**	**CV**
Age (years)	56.6 ± 4.9		65.1 ± 4.9		77.6 ± 4.9	
DBH (cm)	18.6 ± 3.8	20.4%	20.9 ± 3.4	16.2%	23.7 ± 3.2	13.5%
Height (cm)	15.9 ± 3.3	20.8%	17.9 ± 3.0	16.8%	20.5 ± 2.8	13.7%
Volume (m^3)	193.4 ± 62.0	32.1%	239.6 ± 61.4	25.6%	291.8 ± 57.0	19.5%
Deciduous volume (m^3)	16.0 ± 8.2	51.3%	20.1 ± 9.0	44.8%	25.8 ± 9.6	37.2%
Soil C (kg C ha^{-1})	88,745 ± 26,299	29.6%	89,535 ± 26,417	29.5%	92,683 ± 25,485	27.5%
Tree C (kg C ha^{-1})	62,334 ± 19,106	30.7%	75,443 ± 18,025	23.9%	89,665 ± 16,129	18.0%
Ground veg C (kg C ha^{-1})	2,110 ± 261	12.3%	2,107 ± 247	11.7%	2,105 ± 235	11.1%
NEP (g C m^{-2} yr^{-1})	116.7 ± 64	54.8%	113.8 ± 64	56.2%	74.7 ± 62	83.0%
